# Tirzepatide, a dual incretin analog, is a boon in metabolic syndrome: an editorial

**DOI:** 10.1097/MS9.0000000000001782

**Published:** 2024-02-05

**Authors:** Mainak Bardhan, Pooja Gokhale, Priyanka Roy, Tithishri Kundu, Ayush Anand

**Affiliations:** a Miami Cancer Institute, Baptist Health South Florida, Miami, USA; b Shree Chanakya Education Society’s Indira College of Pharmacy, Pune; c Deputy Chief Inspector of Factories/ Deputy Director (Medical) and Certifying Surgeon, Directorate of Factories, Department of Labour, Government of West Bengal; d Department of Pharmacology, Manipal Tata Medical College Jamshedpur, Manipal Academy of Higher Education, Manipal, Karnataka, India; e B. P. Koirala Institute of Health Sciences, Dharan, Nepal

*Dear Editor*,

Metabolic syndrome is characterized by various metabolic abnormalities, that is, abdominal obesity, insulin resistance, hyperlipidemia, hypertension, and metabolic dysfunction-associated fatty liver disease (MAFLD), and carries cardiovascular risk requiring early diagnosis and management. Metformin, statin, renin-angiotensin-aldosterone system inhibitor, and glucagon-like peptide 1 (GLP-1) receptor agonists have been utilized as pharmacological options in managing metabolic syndrome. Tirzepatide is a novel dual incretin receptor agonist that activates both glucagon-like peptide (GLP-1) and glucose-dependent insulinotropic polypeptide (GIP). GLP-1, has dual uses, that is, US Food and Drug Administration approved management of type 2 diabetes mellitus (T2DM) and obesity. In this study, we present a brief summary of the literature regarding the utilization of tirzepatide to treat all components of metabolic syndrome and propose the use of tirzepatide in metabolic syndrome.

## Tirzepatide for diabetes

Tirzepatide leads to adiponectin release and ultimately improves insulin sensitization and lowers glucose levels in type 2 diabetes. The SURPASS (1-4) trials demonstrated the effectiveness of tirzepatide compared to placebo and other antidiabetic agents, that is, semaglutide, insulin degludec (as an adjunct to metformin), and insulin glargine, respectively^1–4^. SURPASS-5 concluded that when added to titrated insulin glargine therapy, tirzepatide significantly reduced HbA1c levels at week 40 than placebo^5^. Therefore, tirzepatide can be a feasible option to treat type 2 diabetes.

## Tirzepatide for obesity

In SURPASS^1,2,5^ and SURMOUNT-1 trials^6^ and SURPASS J-mono sub-trial^7^, tirzepatide showed a substantial decrease in body weight. Compared to the bodyweight escalation observed with insulin degludec, tirzepatide showed a weight loss in the SURPASS-3 trial^3^. It was hypothesized that agonism of GIP and GLP-1 in the CNS might be responsible for weight loss with tirzepatide^8^. Thus, tirzepatide can be considered a viable option for treating obesity.

## Tirzepatide for hyperlipidemia

A 26-week study concluded that tirzepatide once-weekly administration decreased triglyceride, apolipoprotein B, and apolipoprotein C-III in a dose-dependent manner, thus improving atherogenic lipoprotein profile. Tirzepatide also increased serum preheparin lipoprotein lipase. A decrease in the low-density lipoprotein particles, and triglyceride-rich lipoprotein particles, was also reported compared to placebo and dulaglutide^9^. Therefore, tirzepatide can be an effective treatment for hyperlipidemia.

## Tirzepatide and its effects on blood pressure

Tirzepatide has demonstrated a modest decrease in hypertension in all the SURPASS trials^1–4^. SURPASS-2, SURPASS-3, SURPASS-5, and SURMOUNT-1 trials reported systolic and diastolic BP reduction with tirzepatide^2,3,5,6^. In SURPASS-4 trial, mean systolic and diastolic BP were reduced with tirzepatide and, in comparison, increased with insulin glargine^4^. In the SURPASS-1 trial, the systolic BP was decreased by 4.7 mm to 5.2 mmHg, though the diastolic BP did not change significantly^1^.

## Tirzepatide for MAFLD

MAFLD is often a component of metabolic syndrome. Recently, in SURPASS-3 sub-trial, tirzepatide has demonstrated a significant reduction in liver fat content of 8.09%, compared to a 3.38% decrease seen with insulin degludec^10^. The treatment difference was (−4.71%) in tirzepatide compared to insulin degludec in this study^10^. Hence, it is evident that tirzepatide can cause a higher reduction in liver fat content and thus can be used in MAFLD. But a direct comparison of efficacy between tirzepatide and insulin degludec in a clinical trial is not available till now.

## Tirzepatide and cardiovascular risk

Patients suffering from metabolic syndrome have an augmented cardiovascular risk. In a meta-analysis, tirzepatide did not increase the chance of major adverse cardiovascular events (MACE), that is, unstable angina, stroke, myocardial infarction, and cardiovascular death. Furthermore, tirzepatide has improved biomarkers associated with MACE, that is, leptin, YKL-40, and intracellular adhesion molecule-1 (ICAM-1) levels compared to GLP-1 agonist dulaglutide in a phase 2 study^11^. But further data regarding the effect of this drug on cardiovascular outcomes is needed. The SURPASS CVOT trial, which will be completed in October 2024, might play a significant role.

## Conclusion

Therefore, we conclude that tirzepatide may soon become a revolutionary treatment in managing metabolic syndrome due to its beneficial effects on diabetes mellitus, obesity, hyperlipidemia, hypertension, and MAFLD. Furthermore, it does not increase and might improve cardiovascular risk associated with metabolic syndrome. Further research regarding its efficacy is required, as is more data regarding possible adverse effects.

## Ethical approval

Ethical approval is not applicable for this letter to editor.

## Consent

Informed consent is not applicable for this letter to editor.

## Sources of funding

The authors did not receive any funding for this work.

## Author contribution

M.B., P.G., P.R., T.K., and A.A.: conceptualization, supervision, writing – original draft, and writing – review and editing.

## Conflicts of interest disclosure

The authors do not have any conflicts of interests to declare.

## Research registration unique identifying number (UIN)

Not applicable.

## Guarantor

Mainak Bardhan.

## Data availability statement

Not applicable.

## Provenance and peer review

Not commissioned, externally peer-reviewed.
